# Speed Effect Analysis Using the CFA Framework

**DOI:** 10.3389/fpsyg.2019.00239

**Published:** 2019-02-14

**Authors:** Karl Schweizer, Siegbert Reiß, Xuezhu Ren, Tengfei Wang, Stefan J. Troche

**Affiliations:** ^1^Institute of Psychology, Goethe University Frankfurt, Frankfurt, Germany; ^2^Department of Psychology and Behavioral Sciences, Zhejiang University, Hangzhou, China; ^3^School of Education, Huazhong University of Science & Technology, Wuhan, China; ^4^Institut für Psychologie, University of Bern, Bern, Switzerland

**Keywords:** processing speed, normal distribution, structural validity, omissions, model of measurement

## Abstract

The paper outlines a method for investigating the speed effect due to a time limit in testing. It is assumed that the time limit enables latent processing speed to influence responses by causing omissions in the case of insufficient speed. Because of processing speed as additional latent source, the customary confirmatory factor model is enlarged by a second latent variable representing latent processing speed. For distinguishing this effect from other method effects, the factor loadings are fixed according to the cumulative normal distribution. With the second latent variable added, confirmatory factor analysis of reasoning data (*N*=518) including omissions because of a time limit yielded good model fit and discriminated the speed effect from other possible effects due to the item difficulty, the homogeneity of an item subset and the item positions. Because of the crucial role of the cumulative normal distribution for fixing the factor loadings a check of the normality assumption is also reported.

## Introduction

The present paper concentrates on the identification of the speed effect obvious in data with a large number of omissions because of not-reached items. The omissions are assumed to originate from the co-action of participants' processing speed and a time limit in testing. It is explored whether the speed effect can be identified by means of confirmatory factor models. Because of the time limit in testing only some participants may reach all items whereas other participants only process subsets of items and, consequently, produce incomplete data. The time limit terminates the cognitive processing irrespective of whether the next response would be correct or incorrect. Since such data differ from what is expected according to the customary models of measurement (Graham, 2006) used in confirmatory factor analysis (CFA), the speed effect is a source of model misfit. Although there are also other sources of omissions (Oshima, 1994), the speed effect appears to be the strongest source—especially in the assessment of abilities.

The speed effect is only one of a number of method effects that may distort measurement. Method effects are sources of systematic variance of observational data besides the systematic variance that is due to the expected source, i.e., the construct actually intended to be measured. The multitrait-multimethod (MTMM) research initiated by Campbell and Fiske (1959) suggests that method effects are more the norm than the exception. However, despite the progress of the methodology, especially of the statistical modeling (Byrne, 2016), the expense for this kind of research is still large, and the major benefit is insight into whether the data show a method effect or not. The improvement of the quality of psychological assessment has to be achieved outside of the multitrait-multimethod framework. Since multitrait-multimethod research is restricted to a specific research design, *post-hoc* investigations focusing on the presence of a specific method effect in given data are outside of its reach.

Omissions pose a special challenge to data analysis: if some individuals omit some of the last items, there is a change of the sample of participants regarding these items. It means that the last items of a sequence of items are completed by another sample than the first items. These samples systematically differ from each other with respect to processing speed and eventually also due to other characteristics. It is possible to replace omissions by means of one of the available imputation methods (e.g., O'Muircheartaigh and Moustaki, 1999; Holman and Glas, 2005; Finch, 2008). Since these methods perform extrapolation on the basis of the available information, the outcome depends on the correctness of the information on the participants' performance. Furthermore, the amount of available information is relevant for the accuracy of imputation. The more information on a participant is missing, the less likely is the correct replacement of omissions.

In this paper we propose an alternative method of treating omissions in the framework of CFA. It is assumed that omissions influence the relationships among the items in such a way that an independent latent variable is necessary to account for the additional systematic variance if treated as incorrect responses in structural investigations. Furthermore, it is assumed that the relationships among items with omissions are so specific that they can be distinguished from relationships among items due to other method effects.

### The Model of Measurement

CFA is mostly conducted on the basis of a one-factor model of measurement. Although there are several one-factor models (Graham, 2006), structural investigations are usually conducted by means of the congeneric model (Jöreskog, 1971). The following equation describes this model:

(1) $$\begin{array}{l} \text{x}=\lambda \xi +\delta \end{array}$$

where the *p*×1 vector **x** represents the centered data, the *p*×1 vector **λ** the factor loadings, the latent variable ξ the latent source of responding and the *p*×1 vector δ the error components. The presence of only one latent variable reflects the assumption of only one systematic source of responding.

Models developed to investigate MTMM data assume two sources of responding (Byrne, 2016). The participants' trait is assumed as the first source and the observational method as the second source. Both sources are assumed to be simultaneously active. These assumptions are represented by the two-factor model of measurement that includes components for the trait and method sources as given by the following equation:

(2) $$\begin{array}{l} {\text{x}}_{\text{scores}}=\lambda_{\text{trait}}\xi_{\text{trait}}+\lambda_{\text{method}}\xi_{\text{method}}+\delta \end{array}$$

where the *p*×1 vector **x**_scores_represents the centered observations that are usually scale-level scores, the *p*×1 vectors **λ**_trait_ and **λ**_method_ the factor loadings, the latent variables ξ_trait_and ξ_method_ the assumed sources of variance in responding and the *p*×1 vector **δ** the error components.

By definition, however, a MTMM design must include several traits and several methods. Therefore, the model of Equation 2 is incomplete. Formal completeness requires that it comprises the contributions of *r* (>1) traits and *s* (>1) methods such that

(3) $$\begin{array}{l} {\text{x}}_{\text{scores}}=\lambda_{\text{traitA}}\xi_{\text{traitA}}+\ldots +\lambda_{\text{traitR}}\xi_{\text{traitR}}+\lambda_{\text{methodA*}}\xi_{\text{methodA*}}\\ \;+\ldots +\lambda_{\text{methodS*}}\xi_{\text{methodS*}}+\delta \end{array}$$

where the letters A to R and A^*^ to S^*^ refer to the considered traits and methods, respectively. Furthermore, it must be assured that only one trait and one method account for one score. Since each one of the *t* (= *r* + *s*) latent variables ξ_*j*_ (*j* = 1, …,*t*) is associated with a set of indicators included in set C_*j*_, factor loadings can be specified by referring to the corresponding set:

(4) $$\lambda_{ij}=\{\begin{array}{c} \text{est}\lambda_{ij}\text{ if indicator }i\in {\text{C}}_{j}\\ 0\text{ otherwise } \end{array}$$

whereλ_*ij*_ is an element of the *p*×*t* matrix **Λ** of factor loadings that includes all individual vectors of Equation 3, *i* (*i* = 1, …, *p*) identifies the indicator, and estλ_*ij*_signifies that this is a parameter to be estimated. An additional important assumption of MTMM models is that traits and methods are independent of each other whereas the individual traits and methods may correlate among each other depending on the specification of the model.

The investigation of the speed effect as proposed in the present paper is not conducted on the scale level that is focused within the MTMM approach but on the item level. Given that the time limit in testing is constant, processing speed is inserted as source of the speed effect into the formal description but not the time limit. The structure of the model of measurement reflecting processing speed corresponds to the reduced version of the model for investigating MTMM data (Equation 2):

(5) $$\begin{array}{l} {\text{x}}_{\text{items}}=\lambda_{\text{construct}}\xi_{\text{construct}}+\lambda_{\text{processing }-\text{speed}}\xi_{\text{processing }-\text{speed}}+\delta \end{array}$$

where the *p*×1 vector **x**_items_ represents the centered observations that are usually binary data, the *p*×1 vectors **λ**_construct_ and **λ**_processing−speed_ the factor loadings, the latent variables ξ_construct_ and ξ_*proces*sin*g*−*speed*_ the latent sources of responding and the *p*×1 vector **δ** the error components.

Since the construct (i.e. the ability actually intended to be measured) contributes to completing all items, C_construct_ includes all items. In contrast, only the subset of items showing omissions can be expected to reflect processing speed and, therefore, should be included in the set of the processing-speed latent variable C_processing−speed_. This gives the following specification:

(6) $$\lambda_{ij}=\{\begin{array}{l} est\lambda_{\text{ij}}if\{\begin{array}{l} \text{j }refers\;to\;construct\\ \text{j }refers\;to\;proces\;\sin\;g\;-\;speed\;\wedge \;item\text{ i}\in \\ \;C_{proces\;\sin\;g-speed} \end{array}\\ 0\;otherwise\; \end{array}$$

The definition of C_processing−speed_ eventually needs further specification if omissions due to other sources or random responses are suspected.

The model of measurement according to Equations 5 and 6 can be expected to work well in investigations aiming at the identification of the speed effect. However, it is unlikely to do well in discriminating between different method effects since different method effects may lead to the same pattern of constrained and free factor loadings as outlined in the next paragraph.

### The Fixation of Factor Loadings and its Consequences

The fixation of factor loadings facilitates to distinguish between different sources of responding especially when these sources lead to the same subsets of factor loadings that are set free for estimation respectively are set equal to zero. For example, the speed effect and the effect due to a homogeneous subset of items may involve the same items at the end of the scale. However, they may differ according to the sizes of expected factor loadings; it means the pattern of factor loadings. The speed effect means a gradual increase of the number of omissions to be represented by increasing numbers serving as factor loadings whereas the other effect requires the representation by equal-sized factor loadings.

The representation of method effects using patterns of factor loadings requires the fixation of factor loadings according to the pattern characterizing the method effect. In the past, fixed parameters have rarely been considered in CFA research; in the rare cases at least the parameters of the error components had to be set free for estimation (Millsap and Everson, 1991; Millsap, 2001). In contrast, in research according to item-response theory (IRT) the use of fixed parameters is quite common. There is the discriminability parameter of IRT models, for example, that is considered to be equivalent to the factor loading of CFA (Lucke, 2005). Constrained discriminability characterizes the Rasch (1960) model, the corresponding one-parameter model (Birnbaum, 1968) and the Rasch model-based linear logistic test model (Kubinger, 2009).

Both free and fixed factor loadings can be used for reproducing the *p*×*p* empirical covariance matrix **S** by the model of the *p*×*p* matrix of variances and covariances **Σ** that is defined as

(7) $$\begin{array}{l} \Sigma ={\Lambda \Phi \Lambda }^{\prime }+\Theta \end{array}$$

where **Λ** is the *p*×*q* matrix of factor loadings, **Φ** the *q*×*q* matrix of the variances and covariances of the latent variables and **Θ** the *p*×*p* diagonal matrix of error variances (Jöreskog, 1970). This model reflects the model of measurement of Equation 1. Free and fixed factor loadings can be expected to do equally well in investigations of model fit if they correspond and all the other characteristics are the same. Only the degrees of freedom should differ because of the constraint of factor loadings.

However, since the expected pattern of factor loadings may only reflect the relationships among the factor loadings appropriately but not their exact sizes, it is necessary to set the variance parameters φ_*ii*_ (*i* = 1, …,*p*) of **Φ** free for estimation. For a demonstration, assume the expected pattern of factor loadings included in the *p*×1vector **λ**_fixed_ of **Λ** specified according to a simple linear function that reflects the relationships among the freely estimated factor loadings of the *p*×1vector **λ**_free_ of **Λ** correctly but not the exact sizes of the factor loadings. In this case there is a constant *c* (*c* > 0) such that **λ**_free_ = c**λ**_fixed_. This leads to the following equalities:

(8) $$\begin{array}{r} \lambda_{\text{free}}\varphi \lambda_{\text{free}}=c\lambda_{f\text{ixed}}\varphi c\lambda_{\text{fixed}}=\lambda_{\text{fixed}}c^{2}\varphi \lambda_{\text{fixed}}\\ =c^{2}\left(\lambda_{\text{fixed}}\varphi \lambda_{\text{fixed}}\right) \end{array}$$

because *c* is a scalar. If *c* is unknown, the free estimation of φ is likely to lead to a better model fit than assigning any value to it except of the correct one that, however, is usually unknown.

### The Representation of the Speed Effect

The representation of the speed effect as part of a confirmatory factor model by fixed factor loadings requires the assignment of numbers to factor loadings that reflect the effect. These numbers have to represent the influence of processing speed on responding that is actually the lack of sufficient processing speed on responding. In contrast, the other source of the speed effect, the time limit in testing, is the same for all items and, therefore, can be ignored.

The influence of processing speed on responding is apparent in the omissions (Oshima, 1994). More specifically, the omissions show a distribution that can be assumed to reflect the distribution of processing speed. Since the frequencies of omissions usually show a non-linear increase up to the last item, it is presumably a cumulative distribution that has to be reflected by the factor loadings.

In the population, processing speed can be assumed to follow a normal distribution because it appears to be due to a multitude of specific sources (Roberts and Stankov, 1999) as also suggested by recent models of cognitive abilities. In the Cattell-Horn-Carroll model, for example, three broad abilities associated with speed are postulated and rooted in several specific abilities (McGrew, 2009).

The standard normal distribution *N*(μ,σ^2^) is a symmetric distribution with zero mean (μ = 0), unit variance (σ^2^ = 1) and the probability density f(*x* |μ, σ^2^):

(9) $$\begin{array}{l} f\left(x|\mu ,\sigma^{2}\right)=\frac{1}{2\pi \sigma^{2}}e^{-\frac{{\left(x-\mu \right)}^{2}}{2\sigma^{2}}} \end{array}$$

The normal distribution of processing speed is likely to deviate from the standard normal distribution since speed cannot be smaller than zero. Furthermore, there is dependency on the time span available for completing the items: the larger the available time span, the farther away the mean of the distribution from zero. Moreover, the distribution of processing speed must not necessarily show unit variance.

Given that processing speed is distributed normally, the factor loadings have to follow the corresponding cumulative distribution function, i.e. the normal ogive. The normal ogive appears to be well reflected by the logistic function (Lord, 1965). The course of the logistic function signifies that the proportion of variance explained by processing speed is likely to increase when moving from one item to the next item until virtually the complete variance is explained by processing speed in the case of a very short time span for completing the items.

Using this function, the factor loading of the *i*th item (*i* = 1,…,*p*) on the processing-speed latent variable λ(*i*) identified by the subscript *processing-speed* is defined as

(10) $$\begin{array}{l} \lambda_{\text{processing}-\text{speed}}\left(i\right)=\frac{e^{i-tp}}{1+e^{i-tp}} \end{array}$$

It includes the parameter *tp* for adjusting the course of the logistic function to the course of the observed distribution. This means that the adjustment to the unkown mean μ of the latent distribution is conducted by means of *tp*. The corresponding factor loadings are referred to as mean-adapted.

Adjustment to the variance σ^2^ of the underlying normal distribution is achieved by means of multiplier *a* (*a* > 0):

(11) $$\begin{array}{l} \lambda_{\text{processing}-\text{speed}}\left(i\right)=\frac{e^{a\left(i-tp\right)}}{1+e^{a\left(i-tp\right)}} \end{array}$$

The corresponding factor loadings are referred to as mean-variance-adapted.

Although the logistic function serves well for representing processing speed in similar applications (Schweizer and Ren, 2013), it is an open question whether the normal distribution really characterizes processing speed. To answer this question, the following procedure is proposed: (1) estimation of model fit using free factor loadings. Given that the dimensionality of the model is correct, free factor loadings can be assumed to provide the best-possible representation of the cumulative distribution of processing speed since in *p* items *p* parameters are estimated. (2) Estimation of model fit using fixed factor loadings. Using factor loadings designed according to the cumulative normal distribution function, only one parameter, i.e., the variance parameter, is estimated. This means that the degree of model fit to a considerable degree depends on the correctness of the cumulative normal distribution function reflected by the logistic function that is used for fixing the factor loadings. (3) Comparison of the fit results. We hypothesize that corresponding degrees of model fit are observed for free and fixed factor loadings if the true distribution of processing speed is the normal distribution and the fixed factor loadings are specified accordingly.

For the comparison, we focus on the discrepancy function of maximum likelihood estimation F_ml_ that is used for estimating the model parameters assumed to be included in **θ** (not to be confused with **Θ** of Equation 7) since it is basic to many test statistics (Deng et al., 2018) and also an important ingredient of many fit indices. The fixation of the factor loadings leads to a change of **θ**. To signify the change, λ_e_ is added as subscript in the case of the estimation of factor loadings and λ_fh_ in the case of fixation. The formalized hypothesis of correspondence reads

(12) $$\begin{array}{l} {\text{F}}_{\text{ml}}\left(\theta_{\lambda_{\text{e}}}\right)={\text{F}}_{\text{ml}}\left(\theta_{\lambda_{\text{fh}}}\right) \end{array}$$

This hypothesis may be investigated using the chi-squares associated with the discrepancy function. The chi-squares can be compared by the chi-square difference test since they show different degree of freedom. A disadvantage of this comparison is that in the model with free factor loadings the variance parameter is set equal to one and in the model with fixed factor loadings it is free for estimation. Although it is possible to fix the factor loadings of one manifest variable instead of the variance parameter to overcome this discrepancy, we abstain from this possibility because different selections for the fixation are likely to lead to different results.

Another way of overcoming this disadvantage requires the modification of the model with free factor loadings: the factor loadings are estimated in the first step, and the estimates are used for fixing the factor loadings in the second step. Furthermore, the variance parameter is set free for estimation so that both versions of the model include fixed factor loadings and a free variance parameter. The change of the originally free factor loadings is identified by replacing the subscript λ_e_ and by the subscript λ_fe_. It enables to state the hypothesis in a different way:

(13) $$\begin{array}{l} {\text{F}}_{\text{ml}}\left(\theta_{\lambda_{\text{fe}}}\right)={\text{F}}_{\text{ml}}\left(\theta_{\lambda_{\text{fh}}}\right) \end{array}$$

with λ_fe_ = λ_e_ of Equation (12). The disadvantage of this Equation is the correspondence of the degrees of freedom. However, it is no disadvantage if CFI is used for the comparison of models, which depends on the discrepancy function and is recommended for such comparisons (Cheung and Rensvold, 2002).

If the outcome of testing does not indicate a difference, there is no evidence contradicting the assumption of the normal distribution of processing speed. Although it does not exclude that another distribution may do equally well, there is sufficient reason for staying with the assumption.

### The Representation of Alternative Effects

This section serves the preparation of an investigation addressing the question whether patterns employed for representing specific method effects can show such a degree of specificity that model fit discriminates between correct and incorrect patterns. Given the pattern reflecting the speed effect, possible alternative effects are the difficulty effect, the homogeneity effect and the item-position effect among others.

The difficulty effect ascribes omissions to the difficulties of the items. All kinds of difficulty effects have been reported in recent years (e.g., Undorf and Erdfelder, 2013). In the present case it is argued that some or all participants skip items without responding if they find themselves in the situation of being unable to provide the correct response. Since the items of traditional scales are arranged according to their difficulties, the argument that the difficulties of items lead to omissions gives rise to the following expectation: the closer an item is arranged to the end of the sequence of items, the larger the number of omissions. In this case the numbers, which may serve as factor loadings, must reflect the item difficulties. This can be realized for item *i* [*i* = (*p* – *m* + 1), …,*p*] by computing the difference between one and the probability of a correct response:

(14) $$\begin{array}{l} \lambda_{\text{difficulty\_effect}}\left(i\right)=1-\mathrm{Pr}\left(x_{i}\text{ correct}\right) \end{array}$$

where *x*_*i*_ represents the response to item *i* and *m* the number of items showing omissions.

We denote the consequence of a special case of overall inhomogeneity of a scale as homogeneity effect. In this special case, a subset of items shows higher correlations among each other than with other items of the scale. Such an effect may be due to a measurement characteristic shared by the items of the subset. For example, these items are constructed according to the same rule. In this case, the subset of homogeneous items may necessitate the representation by its own latent variable. If the overall inhomogeneity is due to similar difficulties of the items of the subset, the resulting latent variable is referred to as difficulty factor (McDonald and Ahlawat, 1974). Equal-sized numbers can be expected to serve well for representing this similarity among items. Therefore, the factor loading of item *i* [*i* = (*p* – *m* + 1), …, *p*] λ_homogeneity_effect_ is set equal to constant *c* (*c* > 0):

(15) $$\begin{array}{l} \lambda_{\text{homogeneity\_effect}}\left(i\right)=c \end{array}$$

Finally, the item-position effect denotes the dependency of the item statistics on the positions of the items. These statistics change when the position of an item is changed. It is apparent in the position-related increase of the relative amount of true variance (Knowles, 1988). The position-related variance component is usually represented by an additional latent variable with fixed factor loadings. The fixed factor loadings of λ_position_effect_ usually follow the quadratic function f_quadratic_. The factor loading of item *i* [*i* = (*p* – *m* + 1), …,*p*] is given by

(16) $$\begin{array}{l} \lambda_{\text{position\_effect}}\left(i\right)={\text{f}}_{\text{quadratic}}\left(i\right)/c \end{array}$$

where *c* is selected to limit the sizes of the largest factor loading to one.

**Figure 1:**
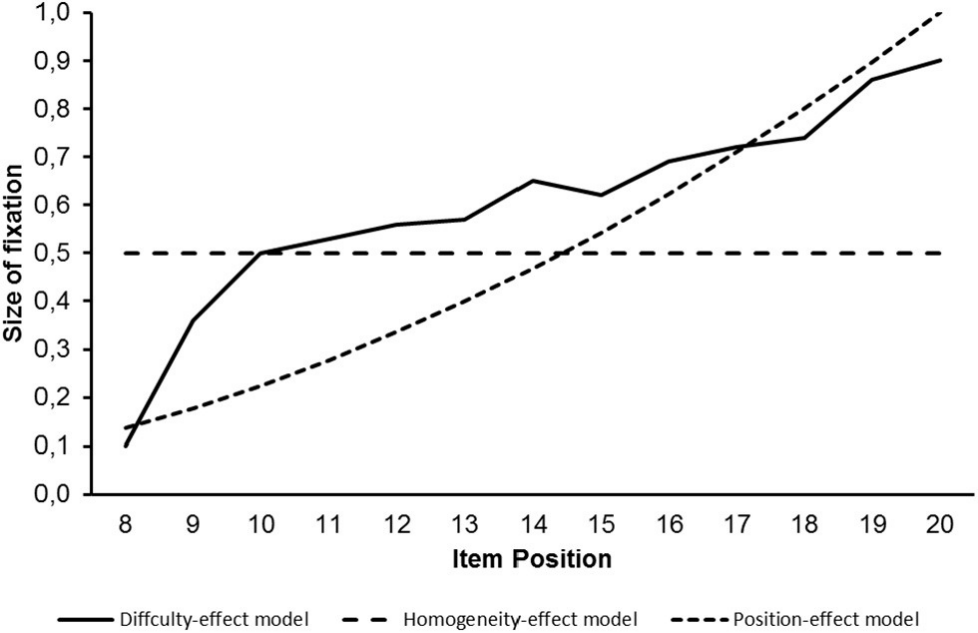
Size of factor loading on the second latent variable for difficulty effect, homogeneity effort and position effect models as curves.

Figure 1 provides curves as illustrations of the factor loadings to be used for the identification of effects due to item difficulty, subset homogeneity and item position.

These curves differ from each other. The differences between the curves for the effects due to item difficulty and item position on one hand and the curve due to sub-set homogeneity on the other hand are especially large.

### Objectives

We examined several objectives of relevance for CFA of data showing omissions that originate from the co-action of a time limit in testing and insufficient processing speed. The first objective was to investigate whether the inclusion of a second latent variable for representing (lack of) processing speed into the confirmatory factor model, referred to as speed-effect model, would lead to improved model fit. This investigation was to be conducted without making distributional assumptions. The second objective was to investigate whether the representation of processing speed by the cumulative normal distribution and fixing the factor loadings by means of the logistic function served as well as the distribution-free representation of processing speed. Finally there was the objective to investigate whether the speed-effect model yielded a better fit in data showing the speed effect than models specified for identifying effects due to item difficulty, the homogeneity of a subset of items and the item positions. This objective required the fixation of factor loadings according to the distribution of processing speed as precondition.

An issue of importance was the selection of the data type for the statistical investigation. While many method studies used simulated data, we decided to use real data. One reason to opt for real data was that simulated data were expected to provide no new insight regarding the second and third aims since models of measurement constructed according to the models used for data generation were unlikely to fail. Another reason was to generate results that would be of special relevance for applied research where the distribution of data might not be known.

## Method

### Sample

In order to have a large set of real data, the data of two samples of university students were combined. The two samples were recruited several years after each other from the same local population of university students using the same incentives. The first sample consisted of 235 university students and the second of 287 university students. Four students were excluded because they did not complete the items or produced incorrect responses only so that the investigated sample included the data of 518 participants. The mean age was 23 years (*SD* = 4.6). The percentage of males was 34 percent. They received either course credit or a financial reward for participating in the study.

### Measure

The reasoning scale used for data collection was taken from Horn's (1983) LPS intelligence test battery. It was selected because the recommended time limit was known to prevent a larger number of participants from completing all items even in university students. The scale consisted of 40 items and showed a good quality according to conventional criteria. The items of this scale required the participants to detect errors in sequences of nine numbers or letters constructed according to a more or less complex rule. The time limit was 8 min. There was no instruction that emphasized to work especially fast.

The first 20 items were so easy for university students that virtually all participants were able to solve them and, consequently, a variance of zero characterized most of these items. However, variances larger than zero were necessary for a positive definite covariance matrix as a precondition for CFA. Consequently, the first 20 items were excluded and the structural investigations were based on the items 21–40. Although these items in general showed an increasing degree of difficulty, there were also small deviations from this general trend. For ease in communication, the remaining items are referred to as items 1–20 in the following sections.

### Models

For the first objective, the data were investigated by means of one- and two-factor confirmatory factor models. The one-factor model referred to as *basic model* included one latent variable representing the construct latent variable with factor loadings from all twenty items and the corresponding error components. The factor loadings were set free for estimation whereas the variance parameter of the latent variable was set equal to one. Two two-factor models were constructed according to Equations (5, 6); they differed in how C_processing−speed_ was defined. In the first version, C_processing−speed_ included all items with the exception of the first one since all items showed omissions. In the second version, only the items 8–20 were assigned to C_processing−speed_. The lack of a systematic increase in omissions in the first to seventh items suggested that they were probably due to other reasons than processing speed.

**Figure 2:**
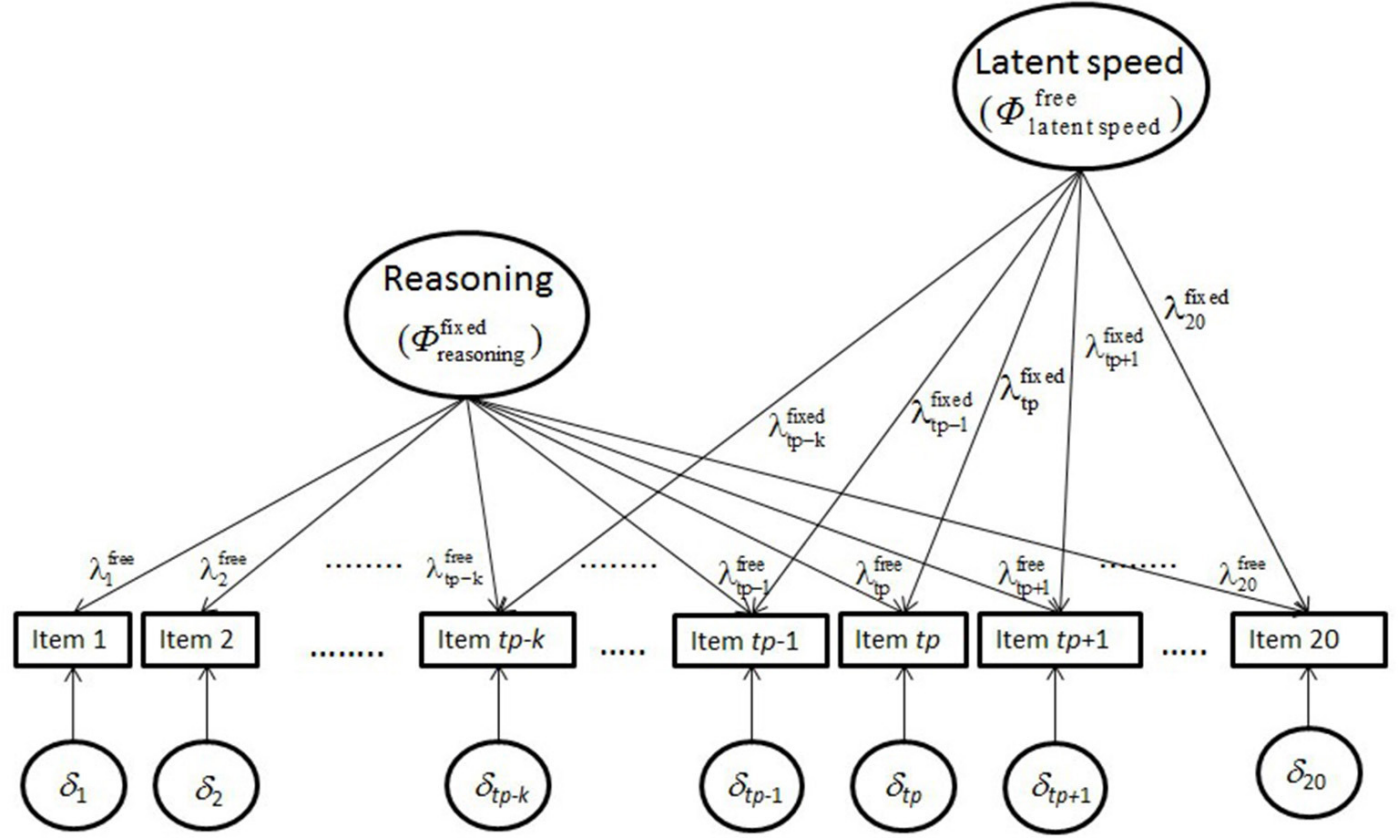
Illustration of the hybrid model including representations of reasoning and latent processing speed.

The second objective required the investigation of the reasoning data by several hybrid two-factor models. In the hybrid models, the factor loadings on the construct latent variable were free for estimation and the factor loadings on the processing-speed latent variable were fixed. There were the hybrid two-factor model with factor loadings according to the mean-adapted normal distribution (Equation 10) and the hybrid two-factor model with factor loadings according to the mean-variance-adapted normal distribution (Equation 11). In these models the variance parameter of the construct latent variable was set equal to one whereas the variance parameter of the processing-speed latent variable was free for estimation. An illustration of this hybrid model is provided by Figure 2.

The parameters of this model include a superscript that indicates whether they are estimated (“free”) or constrained according to the logistic function (“fixed”). Some subscripts of parameters indicating the position of the item have been replaced to signify the position with respect to the *tp* of the logistic function starting with the first numerically noticeable effect of latent processing speed at *tp*-*k*.

**Figure 3:**
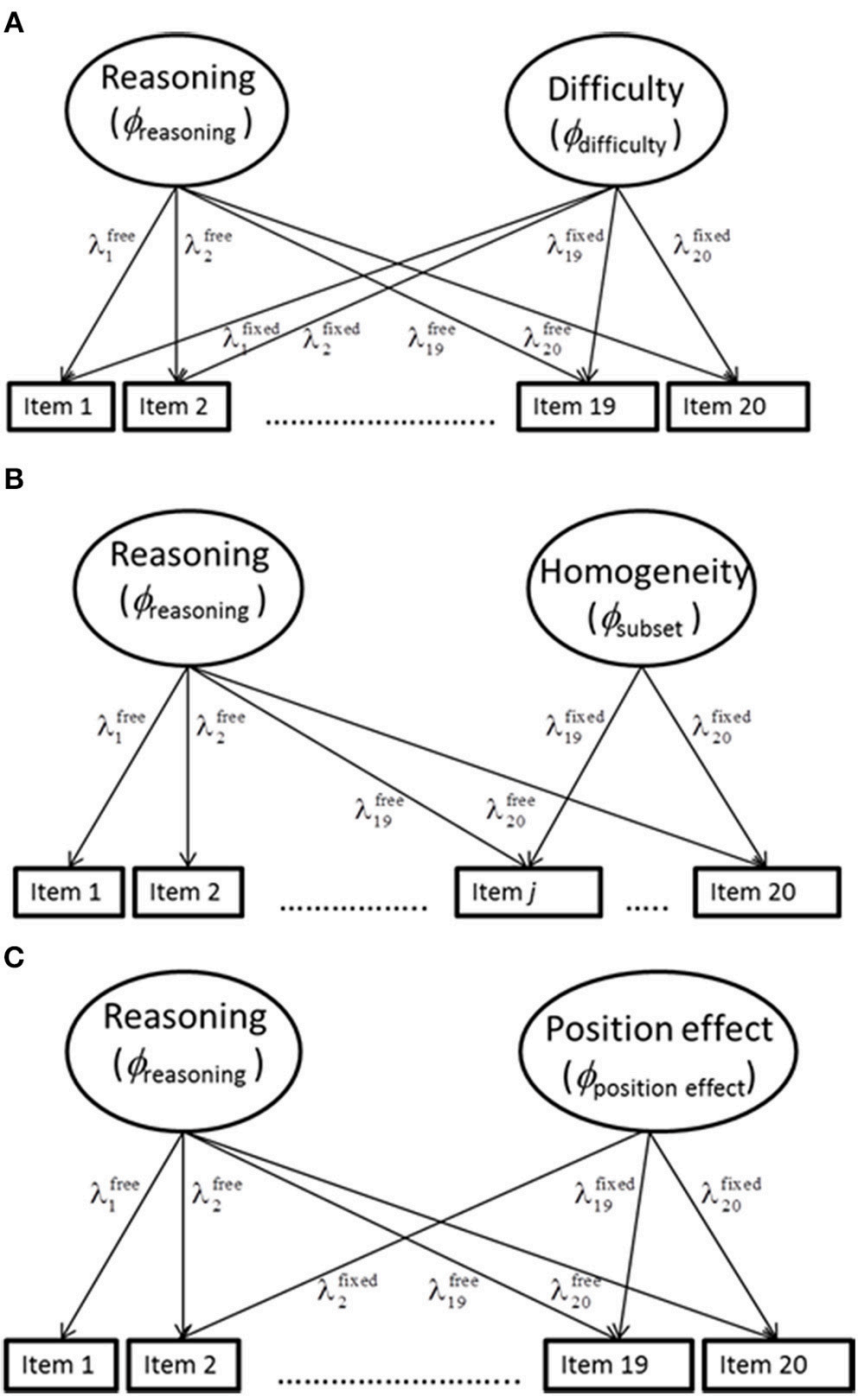
Illustration of hybrid models for representing reasoning together with the difficulty **(A)**, homogeneity **(B)**, and position **(C)** effects. In the models with the additional lateral variable for representing homogeneity, item *j* is the first item of the homogeneous subset. In the model with the additional latent variable for representing the position effect, only the first item shows no cross-loading.

The third objective requiring the comparison with alternative method effects was also investigated by hybrid two-factor models. They showed the same structure as the hybrid models described in the previous paragraph. However, the numbers for constraining factor loadings on the second latent variable were computed according to Equations (14–16) to represent the difficulty effect, the homogeneity effect and the item-position effect, respectively. These models were referred to as difficulty-effect model, homogeneity-effect model and position-effect model in corresponding order. Figure 3 provides illustrations of these hybrid models.

In the difficulty-effect model (A) all items load on both latent variables, in the homogeneity-effect model (B) only the items *j* to 20 show cross-loadings and in the position-effect model (C) cross-loadings characterize all items with the exception of the first one.

### Statistical Analysis

Model parameters and fit indices were estimated using the robust maximum likelihood estimation method by means of LISREL (Jöreskog and Sörbom, 2006). Because of the binary nature of the data, tetrachoric correlations served as input to CFA. Since the matrix of tetrachoric correlations proved to be not positive definite, the ridge option automatically increased the entries of the main diagonal from 1.0 to 1.1. The LISREL code, the matrix of tetrachoric correlations and the asymptotic matrix are provided as Supplementary Materials. For evaluating model fit the following list of fit indices and criteria (in parenthesis) were used: χ^2^,RMSEA (≤0.06), SRMR (≤0.08), CFI (≥ 0.95), NNFI (≥0.95), and AIC (see DiStefano, 2016). The CFI difference served the comparison of models. According to Cheung and Rensvold (2002) a CFI difference of 0.01 can be was considered as a substantial difference. Because the CFI results suggested that there may be a ceiling effect, the chi-square difference was considered additionally in one case.

## Results

### Description of Omissions

**Figure 4:**
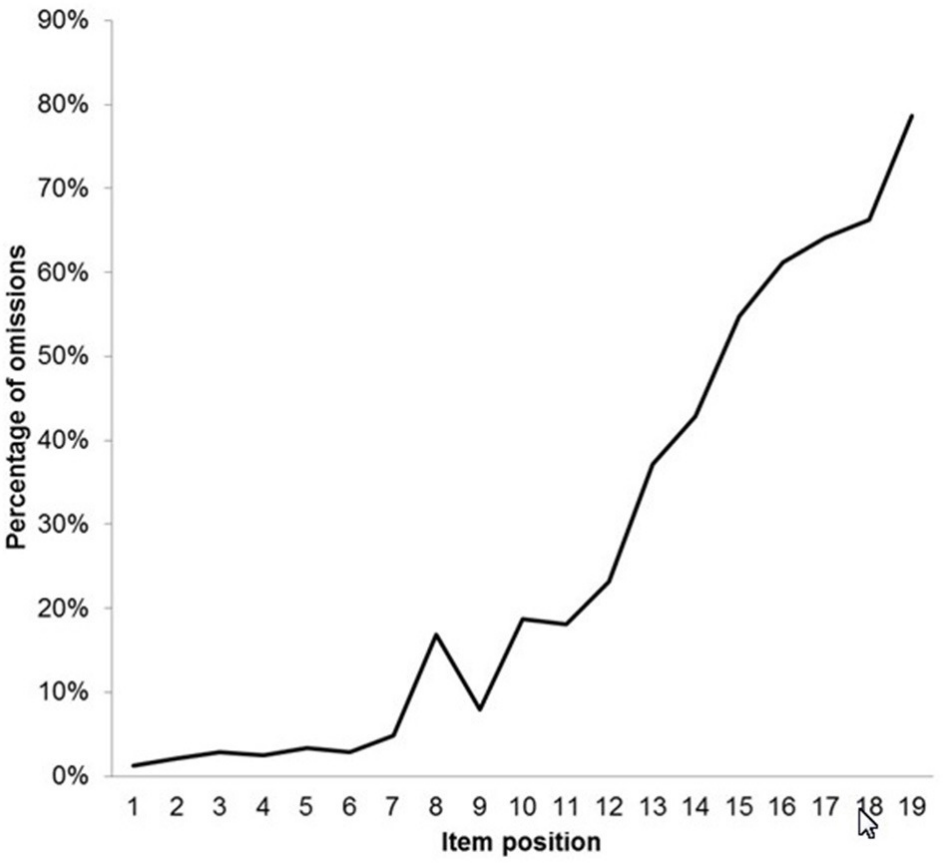
Percentages of omissions for the items of the reasoning scale presented as a curve.

In order to specify the representation of processing speed, it was necessary to examine the frequency distribution of omissions. Figure 4 provides an illustration of the frequency distribution of omissions as a curve.

The curve revealed that all items showed omissions. In the first seven items the level of omissions stayed more or less constant. Starting from item 8 the steepness of the curve increased and followed an almost monotonically increasing course. The omissions preceding the change of the degree of steepness around item 8 suggested that processing speed was presumably not the only source of omissions. Items 1–8 had *p*-values ranging from 0.95 to 0.69 with a mean of slightly over 0.86 while items 9–20, with the one exception of items 14 and 15 which were reversed (0.35 and 0.38, respectively), were monotonically decreasing from 0.64 to 0.10. The change in the course at item 8 was taken as indication of the onset of the influence of speed effect.

### Results Regarding the First Objective

To investigate whether a latent variable can represent the speed effect as part of the confirmatory factor model, one- and two-factor models were investigated. The fit statistics for these models are included in Table 1.

**Table 1:** Fit statistics observed for the models of measurement with free factor loadings (*N* = 518).

**Type of model**	**χ^2^**	**df**	**χ^2^/df**	**RMSEA**	**SRMR**	**CFI**	**NNFI**	**AIC**
One factor	632.0	170	3.71	0.073	0.140	0.952	0.946	712.0
Two factor A^a^	476.1	151	3.15	0.065	0.140	0.966	0.957	594.1
Two factor B^b^	210.5	157	1.34	0.026	0.070	0.994	0.993	316.5

a *All manifest variables with the exception of the first one load on the processing speed latent variable*.

b *Only the last 13 items load on the processing speed latent variable*.

The one-factor model with all factor loadings freely estimated showed good model fit only according to CFI. The NNFI statistic additionally signified good model fit if there was a second latent variable with items 2–20 freely loading on this second latent variable (Two-factor model A). If only items 8–20 loaded on the second latent variable (Two-factor model B), the model showed overall good model fit.

The Two-factor model B was the only model that showed overall good model fit. Furthermore, the differences between the CFIs of this model and the other models were larger than 0.01. In sum, a second latent variable representing processing speed was necessary for appropriately describing the data.

### Results Regarding the Second Objective

The fit results achieved in investigating the appropriateness of the normal distribution assumption are reported in Table 2. The fit results reported in the first row of Table 2 were obtained for the model with fixed factor loadings on the speed factor, whereby these parameters corresponded to the estimates of factor loadings of the Two-factor model B, and the variance parameter was set free. The investigation of this model was necessary for comparisons with models including factor loadings fixed according to the logistic function. Using customary maximum likelihood estimation, the same model fit could be expected for fixed and free factor loadings. However, using robust maximum likelihood estimation as in this study, a change of some fit statistics could be expected. It was obvious when comparing the last row of Table 1 and the first row of Table 2. For example, the chi-square changed from 210.5 to 220.8.

**Table 2:** Fit statistics observed for the hybrid two-factor models of measurement with fixed factor loadings reflecting different distributions (*N* = 518).

**Type of distribution**	**χ^2^**	**df**	**χ^2^/df**	**RMSEA**	**SRMR**	**CFI**	**NNFI**	**AIC**
No assumption^a^	220.8	169	1.31	0.024	0.070	0.995	0.994	302.8
Mean-adjusted normal (logistic)	232.1	169	1.37	0.027	0.081	0.993	0.993	314.3
Mean-variance-adjusted normal (logistic)	228.5	169	1.35	0.026	0.078	0.994	0.993	310.5

a *The results were obtained by turning the freely estimated factor loadings into fixations (see the last row of Table 1)*.

The results reported in the second row of Table 2 were obtained for factor loadings fixed according to the mean-adjusted logistic function. The third row comprises what was observed after additionally adjusting the multiplier of Equation (11) thought to reflect the variance of the normal distribution. All fit statistics included in the three rows indicated good model fit besides SRMR of the model including the mean-adjusted logistic function. No one of the CFIs differences between the models was substantial. This observation was in line with Equation 13. However, because of the overall good CFI results, we suspected that the CFIs showed a ceiling effect, and additionally considered the chi-square difference. In this case the original model with free factor loadings (two-factor model B in Table 1, χ^2^ 210.5) had to be compared with the models with fixed factor loadings. It showed the better model fit when compared with the model including factor loadings according to the mean-adjusted logistic function (chi-square difference = 21.6, *df* = 12, *p* < 0.05). In contrast, no difference was indicated for the model including factor loadings according to the mean-variance-adjusted logistic function (chi-square difference = 18.0, *df* = 12, ns).

**Figure 5:**
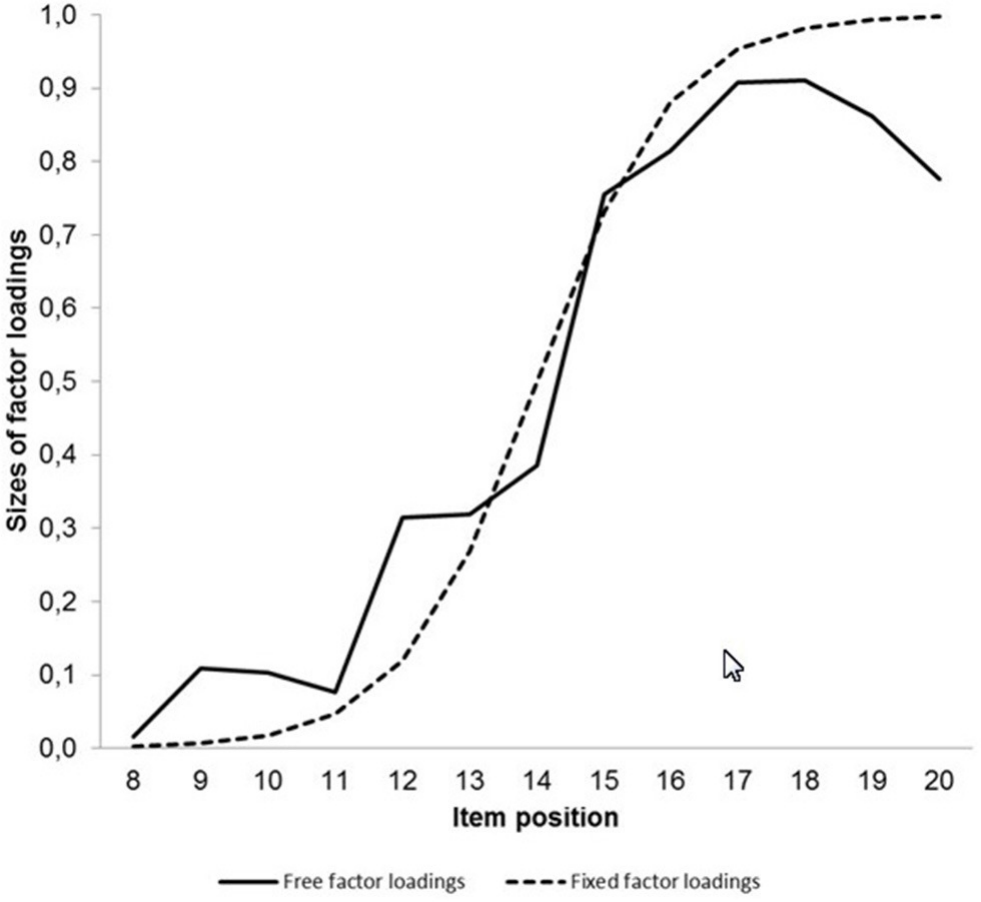
Curves describing the course of the observed free factor loadings and the factor loadings fixed according to the logistic function on the second latent variable representing processing speed.

Figure 5 provides the non-standardized factor loadings on the second latent variable for the model with free factor loadings and the hybrid model including factor loadings according to the logistic function as curves.

The largest discrepancy between the curves was found for the last two items. An attempt to remove these items even led to an increase of model fit.

In sum, neither the CFI difference nor the chi-square difference indicated a difference between the fit results for the model estimated without distributional assumptions and the model with constraints according to the cumulative normal distribution when there was adjustment of the mean and the variance. If there was only adjustment of the mean, the chi-square difference test signified a difference.

### Results Regarding the Third Objective

The fit results for the models specified to represent different method effects are given in Table 3. The first row includes the fit results for the speed-effect model with factor loadings according to the mean-variance adjusted logistic function; these results corresponded to the results reported in the third row of Table 2. The other rows include the results for the difficulty-effect model, the homogeneity-effect model and the position-effect model in corresponding order. All models showed good model fit according to normed chi-square (χ^2^ / df), RMSEA, CFI and NNFI. Again SRMR discriminated between the models. It signified good model fit for the speed-effect model and model misfit for all other models. The numerically best model fit according to each fit index was indicated for the speed-effect model. According to the CFI difference this model fit the data better than the difficulty-effect model and the homogeneity-effect model.

**Table 3:** Fit statistics observed for the models of measurement with the second factor specified to represent a specific effect (*N* = 518).

**Type of effect**	**χ^2^**	**df**	**χ^2^/df**	**RMSEA**	**SRMR**	**CFI**	**NNFI**	**AIC**
Speed effect	228.5	169	1.35	0.026	0.078	0.994	0.993	310.5
Difficulty effect	481.8	169	2.85	0.060	0.166	0.967	0.963	563.8
Homogeneity effect	380.5	169	2.25	0.049	0.167	0.978	0.975	462.5
Position effect	258.1	169	1.53	0.032	0.103	0.991	0.989	340.1

## Discussion

Method effects impair the quality of assessment. The speed effect due to a time limit in testing is just one of them. Since the traditional models of measurement used in psychological assessment assume that there is only one systematic source of responding (Graham, 2006), other systematic sources such as method effects are ignored. However, there is no need for omitting additional sources of responding. The possibility of enlarging the model of measurement by considering additional systematic sources of responding has already been demonstrated in the realm of MTMM analyses (Byrne, 2016).

Numerous MTMM analyses have been conducted in order to identify method effects and have served well for this purpose. These analyses are usually conducted for evaluating the psychometric quality of a scale. The approach used to investigate method effects presented in this paper may be considered as a supplement that may be selected in the case that a *post-hoc* check for the presence of a method effect is necessary. It requires that there is a clear idea of what kind of method effect is to be expected. It can be especially useful for investigating the presence of a speed effect because the speed effect varies as a function of characteristics of the actual sample. For example, a stronger effect can be expected in an older sample than in a younger sample because older people tend to be slower than younger people (Salthouse, 2000).

The models with a second latent variable for representing a method effect can be applied independently of a MTMM design. This independence is achieved by considering effect-specific knowledge. In the case of the speed effect, it is well-known that processing speed influences responding on an achievement test if there is a time limit in testing (Oshima, 1994) and that processing speed as a broad ability in the framework of hierarchical intelligence models can be assumed to be normally distributed (e.g., Roberts and Stankov, 1999). It should be noted that normality is only assumed for a limited range because it cannot extend to values below the zero point of the speed axis.

Although the investigation of whether the second latent variable is necessary can be conducted without specifying the distribution of processing speed, it is good to have knowledge regarding the distribution: (1) knowledge about the distribution facilitates the decision about whether the second latent variable represents a method effect or a specific trait respectively ability because the investigated construct may show a substructure. (2) Such knowledge also provides the opportunity to distinguish between different method effects. A demonstration of this opportunity is provided by the present study. Knowledge about the distribution of the speed effect is of particular importance for separating this effect from other effects such as the difficulty, the homogeneity, or the item-position effects, which can be assumed to follow other distributions.

Deviations from distributional assumptions pose a major problem in confirmatory factor analysis (West et al., 1995; Flora and Curran, 2004) since some frequently used estimation methods presuppose normality of data. However, a deviation from normality seems to be more the norm than the exception (Micceri, 1989). The distributional assumption regarding processing speed differs from the assumptions relevant for the estimation methods. It applies to a latent source that finds its expression in the frequency distribution of omissions in a set of items. For this case we proposed to check whether there is a difference between the fit for freely estimated factor loadings and for factor loading reflecting the normal distribution. Although the result achieved by this method does not exclude that factor loadings based on another distribution do equally well, the method implies that no other distribution does better since it can be assumed that free factor loadings yield the best-possible fit if the assumption regarding the dimensionality is correct.

The overall good model fit may be considered as a limitation of the study since the comparability of models may be impaired, as is suggested by the large sizes of all CFIs. The reason is the use of tetrachoric correlations that are recommended for structural investigations of dichotomous data (Muthén, 1984). Tetrachoric correlations are considered to adequately reflect the relationships between the underlying continuous and normally distributed sources. They can be very large and should be very large if the source is the same, as is in different items of the same scale. Therefore, they can be expected to lead to correlation matrices that are not positive definite, as in the present case. The ridge option (Yuan et al., 2011), used in this study, saves the situation. A consequence of the large correlations is the large size of the CFIs since this fit index includes the comparison of the results for the investigated model with the independence model; the independence model does not reproduce the large correlations and, therefore shows a rather bad model fit.

Another limitation of the present study might be seen in the combination of a specific time limit, of items showing a characteristic pattern of difficulties and of a sample with unique properties. Other combinations may lead to somewhat other distributions of omissions and, consequently, to somewhat different results in structural investigations. Furthermore, other items may additionally stimulate the sources of other effects. Therefore, the generalizability of the present results is limited.

Finally, after listing all the limitations, we like to summarize the major advantages of the described method: it enables the detection of method effects, especially of the effect due to processing speed in combination with a time limit, in a *post-hoc* manner and without resorting to an MTMM design. Furthermore, it implies the control of the influence of method effects on the representation of the construct of interest. Although it was not worked out in detail in this essay, it can be stated that this way the representation of the construct by, a latent variable, is achieved that can be considered as unimpaired by the corresponding method effect.

## Ethics Statement

This study was carried out in accordance with the recommendations of “Ethikrichtlinien, Lokale Ethikkommission des Instituts für Psychologie” with written informed consent from all subjects. All subjects gave written informed consent in accordance with the Declaration of Helsinki. The protocol was approved by the “Lokale Ethikkommission des Instituts für Psychologie.”

## Author Contributions

KS: conceptualized the study and contributed to data analysis and writing. SR: contributed substantially to the writing of the whole article and to data analysis. XR: contributed to the development of the method of analyzing the speed effect. TW: contributed substantially to the writing of the whole article. ST: contributed substantially to conceptualizing and writing of the whole article.

### Conflict of Interest Statement

The authors declare that the research was conducted in the absence of any commercial or financial relationships that could be construed as a potential conflict of interest.
