# Supplementary material for: Speed Effect Analysis Using the CFA Framework
Source: Front Psychol. 2019 Feb 14;10:239. doi: 10.3389/fpsyg.2019.00239 (PMC6382673; doi:10.3389/fpsyg.2019.00239)
Supplement: Supplementary file 1 [file Table_1.DOCX]

**Supplement**

**Model for Speed Effect Analysis** (mean-adapted version)

DA NI=20 NO=518 MA=KM

LA

I1 I2 I3 I4 I5 I6 I7 I8 I9 I10 I11 I12 I13 I14 I15 I16 I17 I18 I19 I20

KM FI=DATA.COR

AC FI=DATA.ACC

MO NX=20 NK=2 TD=DI,FR PH=FU,FI

LK

Reasoning Speed-effect

FR LX 1 1

FR LX 2 1

FR LX 3 1

FR LX 4 1

FR LX 5 1

FR LX 6 1

FR LX 7 1

FR LX 8 1

FR LX 9 1

FR LX 10 1

FR LX 11 1

FR LX 12 1

FR LX 13 1

FR LX 14 1

FR LX 15 1

FR LX 16 1

FR LX 17 1

FR LX 18 1

FR LX 19 1

FR LX 20 1

VA 0 LX 1 2

VA 0 LX 2 2

VA 0 LX 3 2

VA 0 LX 4 2

VA 0 LX 5 2

VA 0 LX 6 2

VA 0 LX 7 2

VA 0.00247 LX 8 2

VA 0.00669 LX 9 2

VA 0.01798 LX 10 2

VA 0.04744 LX 11 2

VA 0.11917 LX 12 2

VA 0.26879 LX 13 2

VA 0.50000 LX 14 2

VA 0.73118 LX 15 2

VA 0.88079 LX 16 2

VA 0.95256 LX 17 2

VA 0.98201 LX 18 2

VA 0.99331 LX 19 2

VA 0.99753 LX 20 2

VA 1 PH 1 1

FR PH 2 2

PD

OU ML SC MI IT=1000 ND=3

**Model for Difficulty Effect Analysis**

DA NI=20 NO=518 MA=KM

LA

I1 I2 I3 I4 I5 I6 I7 I8 I9 I10 I11 I12 I13 I14 I15 I16 I17 I18 I19 I20

KM FI=DATA.COR

AC FI=DATA.ACC

MO NX=20 NK=2 TD=DI,FR PH=FU,FI

LK

Reasoning Difficulty

FR LX 1 1

FR LX 2 1

FR LX 3 1

FR LX 4 1

FR LX 5 1

FR LX 6 1

FR LX 7 1

FR LX 8 1

FR LX 9 1

FR LX 10 1

FR LX 11 1

FR LX 12 1

FR LX 13 1

FR LX 14 1

FR LX 15 1

FR LX 16 1

FR LX 17 1

FR LX 18 1

FR LX 19 1

FR LX 20 1

VA 0.05 LX 1 2

VA 0.15 LX 2 2

VA 0.09 LX 3 2

VA 0.14 LX 4 2

VA 0.04 LX 5 2

VA 0.10 LX 6 2

VA 0.17 LX 7 2

VA 0.15 LX 8 2

VA 0.19 LX 9 2

VA 0.34 LX 10 2

VA 0.32 LX 11 2

VA 0.25 LX 12 2

VA 0.43 LX 13 2

VA 0.44 LX 14 2

VA 0.12 LX 15 2

VA 0.18 LX 16 2

VA 0.15 LX 17 2

VA 0.15 LX 18 2

VA 0.22 LX 19 2

VA 0.46 LX 20 2

VA 1 PH 1 1

FR PH 2 2

PD

OU ML SC IT=1000 ND=3

**Model for Homogeneity Effect Analysis**

DA NI=20 NO=518 MA=KM

LA

I1 I2 I3 I4 I5 I6 I7 I8 I9 I10 I11 I12 I13 I14 I15 I16 I17 I18 I19 I20

KM FI=DATA.COR

AC FI=DATA.ACC

MO NX=20 NK=2 TD=DI,FR PH=FU,FI

LK

Reasoning Homogeneity

FR LX 1 1

FR LX 2 1

FR LX 3 1

FR LX 4 1

FR LX 5 1

FR LX 6 1

FR LX 7 1

FR LX 8 1

FR LX 9 1

FR LX 10 1

FR LX 11 1

FR LX 12 1

FR LX 13 1

FR LX 14 1

FR LX 15 1

FR LX 16 1

FR LX 17 1

FR LX 18 1

FR LX 19 1

FR LX 20 1

VA 0 LX 1 2

VA 0 LX 2 2

VA 0 LX 3 2

VA 0 LX 4 2

VA 0 LX 5 2

VA 0 LX 6 2

VA 0 LX 7 2

VA 0.5 LX 8 2

VA 0.5 LX 9 2

VA 0.5 LX 10 2

VA 0.5 LX 11 2

VA 0.5 LX 12 2

VA 0.5 LX 13 2

VA 0.5 LX 14 2

VA 0.5 LX 15 2

VA 0.5 LX 16 2

VA 0.5 LX 17 2

VA 0.5 LX 18 2

VA 0.5 LX 19 2

VA 0.5 LX 20 2

VA 1 PH 1 1

FR PH 2 2

PD

OU ML SC IT=1000 ND=3

**Model for Position Effect Analysis**

DA NI=20 NO=518 MA=KM

LA

I1 I2 I3 I4 I5 I6 I7 I8 I9 I10 I11 I12 I13 I14 I15 I16 I17 I18 I19 I20

KM FI=DATA.COR

AC FI=DATA.ACC

MO NX=20 NK=2 TD=DI,FR PH=FU,FI

LK

Reasoning Position-effect

FR LX 1 1

FR LX 2 1

FR LX 3 1

FR LX 4 1

FR LX 5 1

FR LX 6 1

FR LX 7 1

FR LX 8 1

FR LX 9 1

FR LX 10 1

FR LX 11 1

FR LX 12 1

FR LX 13 1

FR LX 14 1

FR LX 15 1

FR LX 16 1

FR LX 17 1

FR LX 18 1

FR LX 19 1

FR LX 20 1

VA 0 LX 1 2

VA 0.05263 LX 2 2

VA 0.10526 LX 3 2

VA 0.15789 LX 4 2

VA 0.21052 LX 5 2

VA 0.26315 LX 6 2

VA 0.31578 LX 7 2

VA 0.36842 LX 8 2

VA 0.42105 LX 9 2

VA 0.47368 LX 10 2

VA 0.52631 LX 11 2

VA 0.57894 LX 12 2

VA 0.63157 LX 13 2

VA 0.68421 LX 14 2

VA 0.73684 LX 15 2

VA 0.78947 LX 16 2

VA 0.84210 LX 17 2

VA 0.89473 LX 18 2

VA 0.94736 LX 19 2

VA 1.00000 LX 20 2

VA 1 PH 1 1

FR PH 2 2

PD

OU ML SC IT=1000 ND=3

**DATA.COR** (Tetrachoric correlations)

0.10000D+01 0.11801D+00 0.10000D+01 0.11222D+00 0.29594D+00 0.10000D+01

0.56190D-01 -0.27807D-01 0.85383D-01 0.10000D+01 0.43500D+00 0.12593D+00

0.37299D+00 0.38875D+00 0.10000D+01 0.17444D+00 0.28928D+00 0.39861D+00

0.39989D+00 0.51576D+00 0.10000D+01 0.22185D+00 0.12474D+00 0.17185D+00

0.15363D+00 0.46312D+00 0.23194D+00 0.10000D+01 0.12364D+00 0.33594D+00

0.37768D+00 0.37184D+00 0.38515D+00 0.68939D+00 0.47690D+00 0.10000D+01

0.10853D+00 0.11630D+00 0.37444D+00 0.10394D+00 0.56970D+00 0.42569D+00

0.33062D+00 0.66021D+00 0.10000D+01 0.25654D+00 0.31712D-02 0.93878D-01

0.73812D-01 0.37602D+00 0.21699D+00 0.28952D+00 0.35663D+00 0.33542D+00

0.10000D+01 0.76339D-01 0.13058D+00 0.43376D-01 0.10162D+00 0.68967D-01

0.23699D+00 0.22088D+00 0.54078D+00 0.42297D+00 0.21763D+00 0.10000D+01

0.18877D+00 0.89759D-01 0.97004D-01 0.60728D-01 0.22680D+00 0.15277D+00

0.15361D+00 0.44289D+00 0.38258D+00 0.30812D+00 0.40626D+00 0.10000D+01

0.12361D+00 0.67537D-01 0.29314D+00 0.12168D+00 0.29225D+00 0.50920D+00

0.22242D+00 0.46247D+00 0.46680D+00 0.23282D+00 0.42262D+00 0.37464D+00

0.10000D+01 0.11946D+00 0.16445D+00 0.19305D+00 0.88771D-01 0.33108D+00

0.34120D+00 0.79204D-01 0.44440D+00 0.32995D+00 0.21075D+00 0.35998D+00

0.43887D+00 0.56910D+00 0.10000D+01 0.78428D-02 0.12604D+00 0.62744D-01

-0.10753D-01 0.14037D+00 0.22130D+00 0.22265D+00 0.36106D+00 0.44980D+00

0.22920D+00 0.31985D+00 0.41455D+00 0.48006D+00 0.58582D+00 0.10000D+01

0.12212D+00 0.11788D+00 0.99691D-01 0.87194D-01 0.33808D+00 0.10695D+00

0.17240D+00 0.39454D+00 0.26625D+00 0.16107D+00 0.13622D+00 0.36331D+00

0.45403D+00 0.52401D+00 0.81031D+00 0.10000D+01 0.82097D-01 0.15296D-01

0.77167D-01 0.33121D-01 0.13549D+00 0.20329D+00 0.20523D+00 0.40759D+00

0.25840D+00 0.16205D+00 0.17178D+00 0.36173D+00 0.44110D+00 0.44418D+00

0.72336D+00 0.78392D+00 0.10000D+01 0.78296D-02 0.67037D-01 -0.42740D-03

0.35422D-01 0.16964D+00 0.21560D+00 0.73114D-01 0.29178D+00 0.21268D+00

0.19504D+00 0.18731D+00 0.32279D+00 0.41764D+00 0.41268D+00 0.72795D+00

0.76409D+00 0.90526D+00 0.10000D+01 0.61148D-01 0.12683D+00 0.15126D+00

0.37599D-02 0.14422D+00 0.61945D-01 0.18251D+00 0.44558D+00 0.28841D+00

0.16262D+00 0.59438D-01 0.40108D+00 0.27192D+00 0.36414D+00 0.63379D+00

0.83534D+00 0.81913D+00 0.81780D+00 0.10000D+01 0.89656D-01 -0.10176D+00

0.56833D-01 0.17232D+00 0.16494D+00 0.18140D+00 0.83417D-01 0.38146D+00

0.16012D+00 0.23957D+00 0.20454D+00 0.34527D+00 0.25911D+00 0.33194D+00

0.48668D+00 0.53751D+00 0.72858D+00 0.74269D+00 0.75428D+00 0.10000D+01
